# Differential genetic and biochemical responses of *Beta vulgaris* and *Beta maritima* under salt stress

**DOI:** 10.1186/s12870-025-07010-x

**Published:** 2025-09-02

**Authors:** Rana H. Diab, Walaa A. Abo-Shanab, Reda M. Gaafar

**Affiliations:** Botany and Microbiology Department, Faculty of Science, Tanta University, Tanta, 31527 Egypt

**Keywords:** Salinity stress, Choline monooxygenase, Betaine aldehyde dehydrogenase, Glycine betaine, Gene expression, Osmotic stress, Compatible solutes, Halophyte physiology

## Abstract

**Background:**

Screening and raising salt-tolerant crops on saline land is an affordable and environmentally friendly alternative. This study investigated the physiological and molecular processes in eight *Beta vulgaris* and *Beta maritima* accessions.

**Results:**

A preliminary study was carried out to determine the sublethal concentration of NaCl. The chlorophyll content, fresh and dry weights, relative water content, and potassium and sodium ions of all *B. maritima* and *B. vulgaris* accessions were shown to be significantly decreased under salt stress (250 mM NaCl). The ability of four accessions of *B. maritima* to achieve osmotic adjustment by regulating their ions and water intake as well as producing suitable osmolytes, including total soluble proteins and carbohydrates, proline, and glycine betaine (GB), was linked to their salinity tolerance to avoid toxicity caused by excessive ion buildup, unlike *B. vulgaris*. The overexpression of betaine aldehyde dehydrogenase (*BADH*) and choline monooxygenase (*CMO*) for the production of GB in *B. maritima* under salt stress and the downregulation of the same genes in *B. vulgaris* accession further supported these findings. Changes in total phenols and flavonoid content were also observed in *B. maritima* accessions compared to *B. vulgaris* accessions, which varied significantly.

**Conclusions:**

It was found that, among all the *B. maritima* and *B. vulgaris* accessions, *B. vulgaris* 1 was the most susceptible to salt stress, while *B. maritima* 1 was the most tolerant to salinity stress.

**Supplementary Information:**

The online version contains supplementary material available at 10.1186/s12870-025-07010-x.

## Background

One significant environmental stress that has an impact on agricultural productivity globally is soil salinity. Excessive salt levels impact around 960 million hectares of agricultural land worldwide [1]. Poor irrigation techniques, incorrect fertilizer use, and global temperature change worsen the negative impacts of an annual salinity increase of 1–2% [2]. Sugar beet (*Beta vulgaris* L.) is a member of the Caryophyllales order, which is among the base taxa of core dicots. It is the second largest source of refined table sugar in the world, accounting for 30 to 40%, after sugarcane [3]. Cultivated sugar, fodder, leaf, and garden beets are used to produce molasses, sugar, bioethanol, animal feed, pulp residual, and vegetables [4]. Due to its high sugar content and ability to create valuable byproducts, it holds significant potential to bridge the gap between anticipated and actual sugar production [5]. The sea beet (*Beta maritima* L. ssp. *maritima*) is the halophytic wild ancestor of all domesticated beets. The cultivated varieties migrated from temperate regions to subtropical nations because they require less water and grow more quickly than sugarcane [6, 7]. Particularly found around the beaches of the Mediterranean Sea and the European North Atlantic Ocean, sea beet demonstrates significantly greater resistance to salt during the germination and seedling stages compared to other types of beets [8, 9]. However, various environmental conditions negatively impact beet growth, production, and quality. Due to the low genetic diversity in cultivated beets, wild beets serve as a source of genetic variability for crop development under harsh conditions [10].

In Egypt, salinity is one of the main abiotic stresses restricting crop growth and yield. Sugar beet productivity is significantly impacted by the rising salinization of soil resulting from drought and irrigation with saline water, which is caused by climate change. Therefore, it is essential to choose stress-tolerant beet accessions and understand how varying salinity levels influence the response mechanisms of various beet accessions [11].

Most of the crop development program is thought to benefit from the genetic diversity among the several stress-tolerant accessions [12]. It is thought that the stress-tolerant beet accessions possess the genetic capacity to continue growing under stressful circumstances [13]. The reference genome sequences of sea beet and sugar beet are available and may serve as valuable resources for stress research [14]. Therefore, plant breeders will be able to develop highly salt-tolerant beet genotypes [15, 16], capable of thriving in reclaimed lands adversely affected by salt, sodicity, and poor nutrient availability [17, 18]. Due to its tolerance to salt stress, sugar beet is an excellent option for continuous cultivation in subtropical saline soils [19].

Many plants acquire a variety of suitable solutes, including proline, glycine betaine (GB), sugars, and polyols, when they are under environmental stress. The well-known compatible solutes proline and GB, are essential for the osmotic adjustment process in various species, including higher plants [20]. Higher concentrations of proline and GB can diminish stress damage to plant cells, and salt stress up-regulates the enzymes involved in proline and GB production in various plant species [21]. Proline and GB stabilize membranes, proteins, and enzymes, acting as osmoprotectants to shield higher plants from salt and osmotic stress [22]. One of the most effective solutes is the quaternary ammonium molecule GB, which is found in plants [23]. According to earlier theories, the increased accumulation of GB in plants has a physiological role in reducing osmotic stress, maintaining the water balance between plant cells and their environment, and stabilizing macromolecules under high salt concentrations and cellular dehydration [24, 25]. Some economically significant crops, such as rice (*Oryza sativa*), potatoes (*Solanum tuberosum*), and tomatoes (*Solanum lycopersicum*), are unable to accumulate GB and could be targets for betaine biosynthetic engineering [26]. In plants, animals, and microorganisms, the GB biosynthesis pathway begins with choline and proceeds through a two-step dehydrogenation of choline and the oxygenation of betaine aldehyde, an intermediate that is toxic to plants (natural GB accumulators). The two-step route is catalyzed in animals by betaine aldehyde dehydrogenase (BADH) and in *Escherichia coli* by choline dehydrogenase (CDH) [27]. In higher plants, choline monooxygenase (CMO) and betaine aldehyde dehydrogenase (BADH) catalyze a two-step mechanism that converts choline to GB [28]. Conversely, choline oxidase (COD) is the sole enzyme required for GB production in several microbes, including *Arthrobacter globiformis* and *Arthrobacter panescens* [29].

This work aims to provide a thorough understanding of the effects of severe salt stress on various accessions of sugar beet and halophytic sea beets from multiple habitats at the morphological, biochemical, and molecular levels to understand the mechanisms underlying salt tolerance. Furthermore, investigating the genes involved in GB biosynthesis in sugar beets offers clearer insights into the strategies employed by sea and sugar beets to tolerate stress, which will facilitate the improvement of sugar beet yield under salinity stress.

## Results

### Determination of the lethal and sublethal concentrations of NaCl

Table 1 shows that all *B. maritima* and *B. vulgaris* accessions maintained 100% germination under control and 50 mM NaCl conditions, indicating complete tolerance at low salinity. As salinity levels increased, germination percentages declined across all accessions, with *B. maritima* generally exhibiting higher tolerance than *B. vulgaris*. At 100 mM NaCl, *B. maritima* accessions displayed 80–86.67% germination, while *B. vulgaris* ranged from 60 to 66.67%. At 150 mM, *B. maritima* still maintained moderate germination (53.33–86.67%), but *B. vulgaris* dropped further to 40–60%. A sharp decline was observed at 200 and 250 mM, with germination in *B. maritima* falling to 33.33–46.67% and 6.67–26.67%, respectively, while *B. vulgaris* ranged from 26.67 to 53.33% at 200 mM and 6.67–13.33% at 250 mM. All accessions failed to germinate at 300 mM NaCl, confirming this concentration as the threshold for total inhibition. These findings emphasize the relatively higher salt tolerance of *B. maritima* accessions compared to *B. vulgaris*, particularly under moderate to high salinity levels.

**Table 1:** Effect of different concentrations of NaCl (0, 50, 100, 150, 200, 250, and 300 mM) on the germination percentage of eight different accessions of 14-day-old *B. vulgaris* (L.) and *B. maritima* (L.)

Germination percentage (%)								
NaCl concentration (mM)	*B. maritima 1*	*B. maritima 2*	*B. maritima 3*	*B. maritima 4*	*B. vulgaris 1*	*B. vulgaris 2*	*B. vulgaris 3*	*B. vulgaris 4*
0	100 ± 0.0^a^	100 ± 0.0^a^	100 ± 0.0^a^	100 ± 0.0^a^	100 ± 0.0^a^	100 ± 0.0^a^	100 ± 0.0^a^	100 ± 0.0^a^
50	100 ± 0.0^a^	100 ± 0.0^a^	100 ± 0.0^a^	100 ± 0.0^a^	100 ± 0.0^a^	100 ± 0.0^a^	100 ± 0.0^a^	100 ± 0.0^a^
100	86.67 ± 0.57^b^	86.67 ± 0.57^b^	80 ± 1.00^b^	80 ± 1.00^b^	66.67 ± 0.57^b^	66.67 ± 0.57^b^	60 ± 0.0^b^	66.67 ± 0.57^b^
150	86.67 ± 0.58^b^	80 ± 0.0^c^	53.33 ± 0.58^c^	73.33 ± 0.58^c^	60 ± 0.0^b^	53.33 ± 0.58^c^	40 ± 0.0^c^	53.33 ± 0.58^c^
200	46.67 ± 0.58^c^	46.67 ± 0.58^d^	33.33 ± 1.15^d^	33.33 ± 1.15^d^	53.33 ± 0.58^c^	46.67 ± 0.58^d^	26.67 ± 0.5^d^	26.67 ± 0.5^d^
250	13.33 ± 0.58^d^	6.67 ± 0.58^e^	26.67 ± 0.58^e^	6.67 ± 0.58^e^	13.33 ± 0.58^d^	13.33 ± 0.58^e^	6.67 ± 0.58^e^	13.33 ± 0.58^e^
300	0	0	0	0	0	0	0	0

Values are means of 6 replicates ± standard error. Small identical letters on different columns indicate significant differences between treatments according to duncan’s multiple-range test (*P* ≤ 0.05)

### Effect of salinity stress on chlorophyll content (SPAD) of *B. vulgaris* and *B. maritima*

**Fig. 1:**
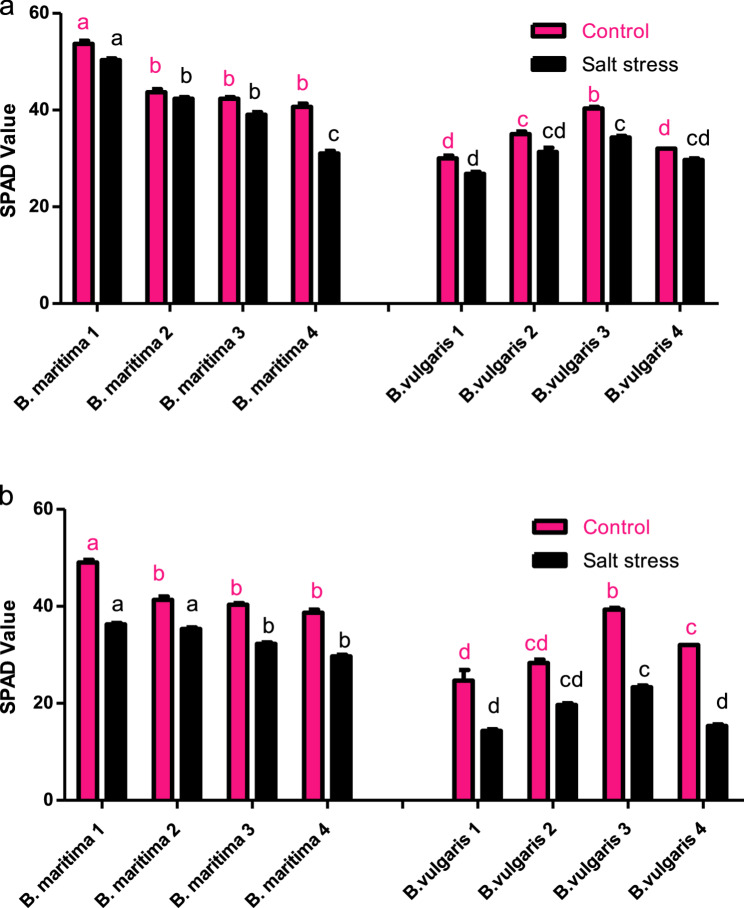
Effect of salinity stress (250 mM NaCl or 25 dS/m) on chlorophyll content (SPAD) of eight different accessions of 63-day-old (**a**) and 70-day-old (**b**) *B. maritima* (L.) and *B. vulgaris* (L.). Values represent means of 6 replicates ± standard error. Small identical letters on different columns indicate significant differences between treatments according to Duncan’s multiple-range test (*P* ≤ 0.05)

The purpose of this study was to compare the effects of 250 mM NaCl on the chlorophyll content of eight accessions of *B. maritima* and *B. vulgaris*, as well as on the chlorophyll content of plants that were nine and ten weeks old after being watered with a 250 mM NaCl solution. Following two and four doses of 250 mM NaCl, as shown in Fig. 1, the results of the SPAD values indicated that the four wild plant accessions (*B. maritima* L.) demonstrate higher tolerance to salt stress than the four cultivated accessions (*B. vulgaris* L.). When *B. vulgaris* plants are nine weeks old, salt stress reduces their chlorophyll content by 7.28–14.86% in comparison to their control plants (Fig. 1a). At ten weeks old, salt stress reduces the chlorophyll content in all four accessions of *B. maritima* – 1, 2, 3, and 4 – by 22.85%, 14.51%, 19.91%, and 23.27%, respectively, compared to the control plants (Fig. 1b). When compared to control plants, salt stress reduces the amount of chlorophyll in all *B. vulgaris* accessions 1, 2, 3, and 4 at ten weeks by 41.91%, 30.56%, 40.68%, and 39.47%, respectively, compared to control plants. These findings indicate that *B. vulgaris* accession 1 is the most vulnerable accession to salt stress conditions, whereas *B. maritima* accession 1 is the most resistant.

### Effect of salinity stress on the growth of *B. vulgaris* and *B. maritima*

**Fig. 2:**
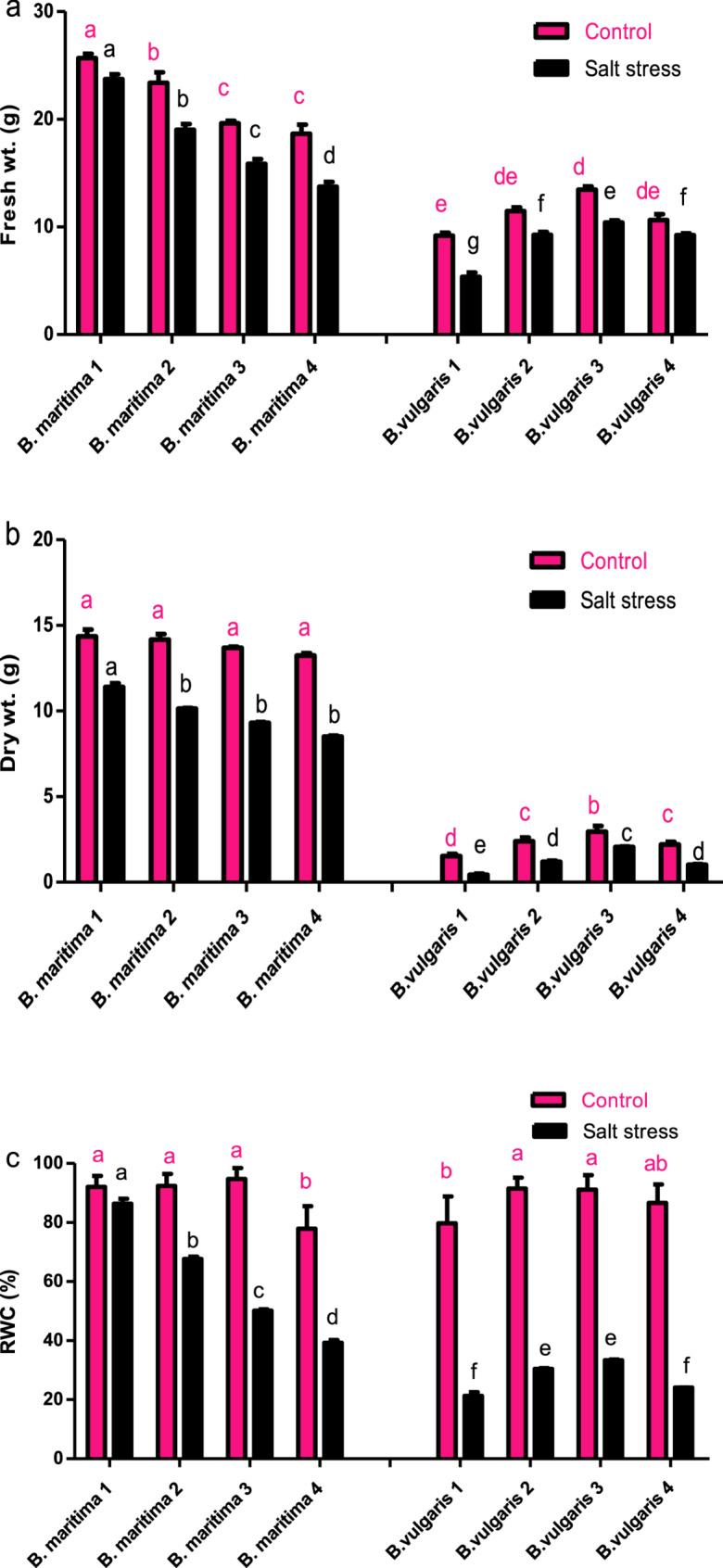
Effect of salinity stress (250 mM NaCl or 25 dS/m) on shoot fresh weight (**a**), shoot dry weight (**b**), and relative water content (**c**) of eight different accessions of 70-day-old* B. maritima* (L.) and *B. vulgaris* (L.). Values represent means of 6 replicates ± standard error. Small identical letters on different columns indicate significant differences between treatments according to Duncan’s multiple-range test (*P* ≤ 0.05)

**Fig. 3:**
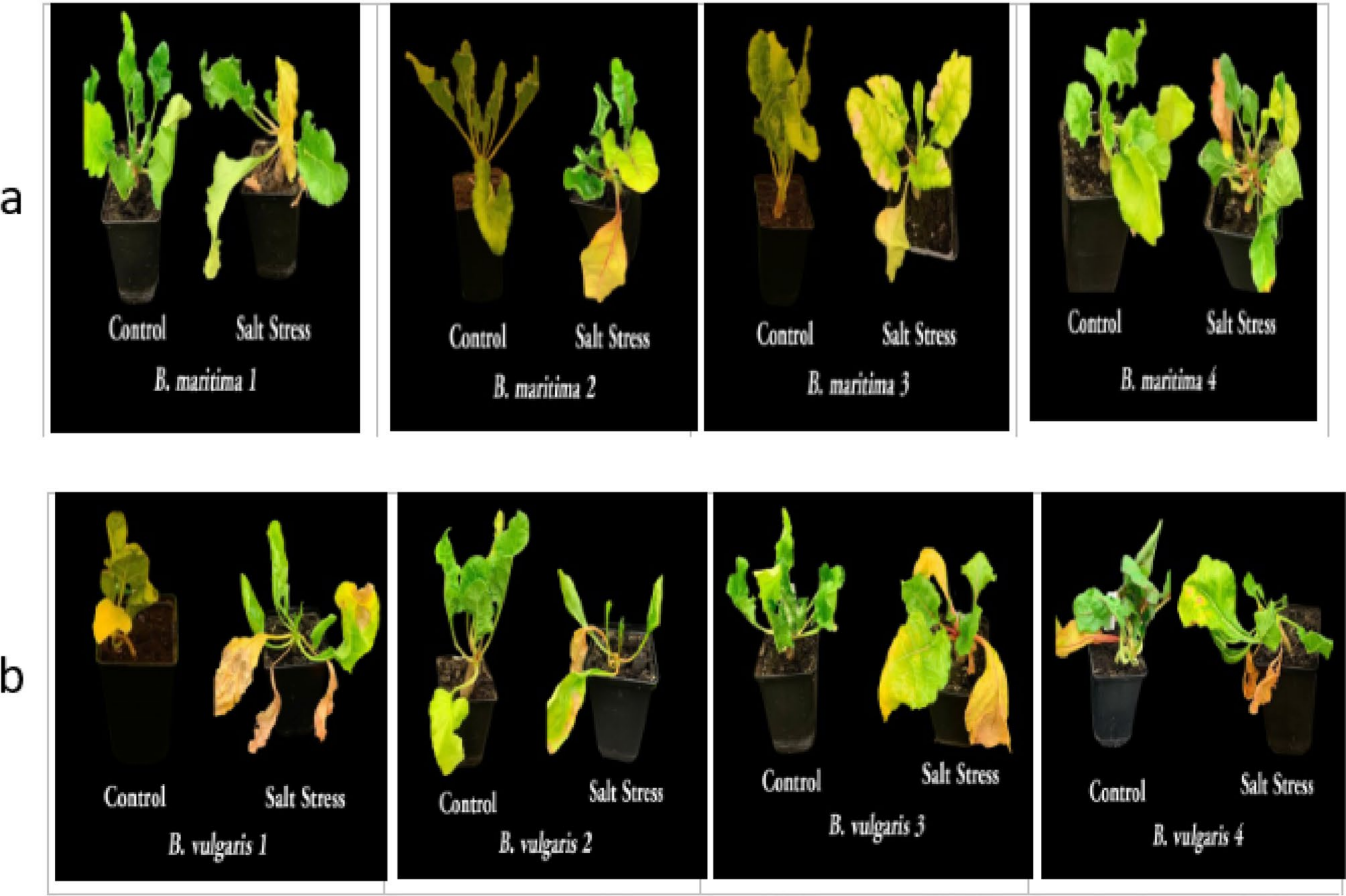
Effect of salinity stress (250 mM NaCl, or 25 dS/m) on the growth of eight accessions of 70-day-old *B. maritima* (L.) (**a**) and *B. vulgaris* (L.) (** b**)

Following the harvesting of 10-week-old plants, measurements were taken of the fresh weight, dry mass, and relative water content of the leaves. As shown in Figs. 2 and 3, our findings demonstrated that salt stress dramatically reduces plant growth compared to the control plants. When *B. vulgaris* accessions 1, 2, 3, and 4 are exposed to salt stress, their fresh weights decrease by 41.63%, 19.18%, 22.79%, and 13.17%, respectively, compared to the control (Fig. 2a); their dry weights decrease by 71.24%, 50.1%, 30.42%, and 53.3%, respectively, compared to the control (Fig. 2b); additionally, the relative water content for all *B. vulgaris* accessions 1, 2, 3, and 4 declines by 78.18%, 66.67%, 64.05%, and 72.16%, respectively, compared to the control (Fig. 2c). Furthermore, the results indicated that *B. maritima* accessions outperformed *B. vulgaris* accessions in all measured parameters when exposed to salt stress. Compared to the control, salt stress reduced shoot fresh weights, dry weights, and relative water content by 6.77%, 20.67%, and 12.50%, respectively, making *B. maritima* 1 the most resistant accession (Fig. 2).

### Effect of salinity stress on compatible osmolytes of *B. vulgaris* and *B. maritima*

**Fig. 4:**
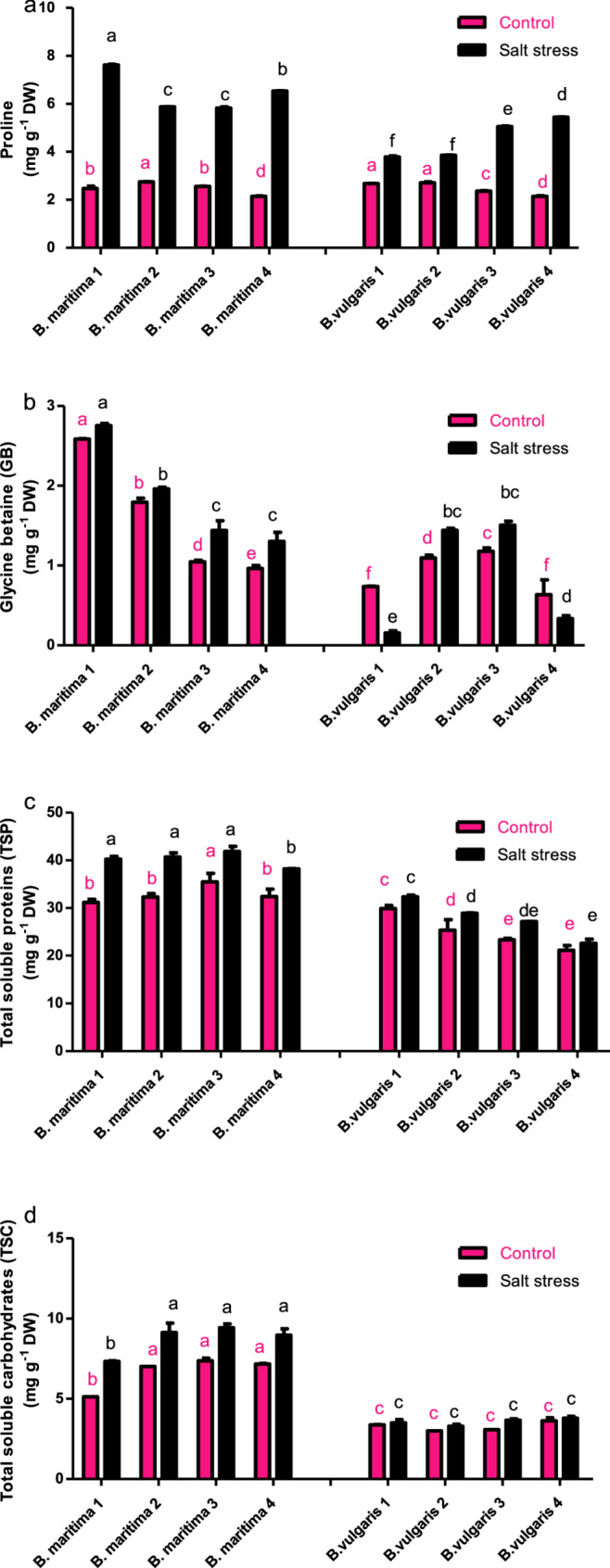
Effect of salinity stress (250 mM NaCl or 25 dS/m) on the content of proline (**a**), glycine betaine (**b**), total soluble proteins (**c**), and total soluble carbohydrates (**d**) of eight different of 70-day-old *B. maritima* (L.) and *B. vulgaris* (L.). Values represent means of 6 replicates ± standard error. Small identical letters on different columns indicate significant differences between treatments according to Duncan’s multiple-range test (*P* ≤ 0.05)

In our study, the proline concentration was determined. The results showed that after exposure to 250 mM NaCl, the proline concentration in the accessions of *B. maritima* 1, 2, 3, and 4 increased by 208.5%, 114.34%, 128.03%, and 204.66%, respectively, compared to their control plants (Fig. 4a). Although *B. vulgaris* 1 had a lower proline concentration than the other salt-treated and control plants, with percentages of 41.47%, 42.07%, 114.27%, and 153.48%, respectively, compared to control, it is considered to be the most sensitive accession to salt stress (Fig. 4a). High proline concentrations are found in *B. maritima* accessions, making them the most tolerant to salt stress. These results indicated that all *B. maritima* accessions, along with *B. vulgaris* 2 and *B. vulgaris* 3, had significantly higher concentrations of GB than the control group when exposed to 250 mM NaCl. These percentages were 6.44%, 9.29%, 37.57%, 34.94%, 31.30%, and 27.68%, respectively (Fig. 4b). However, as shown in Fig. 4b, salt stress reduced the amount of GB in *B. vulgaris* accessions 1 and 4 by 79.18% and 47.36%, respectively, compared to their control plants. Data in Fig. 4c and d demonstrated that the shoots all evaluated accessions generated total soluble proteins and total soluble carbohydrates as osmolytes in response to salt stress. Compared to their control plants, all salt- stressed accessions of *B. maritima* and *B. vulgaris* exhibited high levels of total soluble proteins and carbohydrates. Moreover, *B. vulgaris* 1 and *B. vulgaris* 4 had lower concentrations of total soluble proteins and carbohydrates compared to other identified accessions (Fig. 4c, d). Salinity stress significantly raised the amount of total soluble carbohydrates in *B. maritima* 1 and *B. maritima* 2 compared to other accessions (Fig. 4d). In terms of total soluble carbohydrates and proteins, *B. vulgaris* 3 displayed the highest concentration compared to both its control and other *B. vulgaris* accessions. In contrast, *B. maritima* 1 had the highest concentration of total soluble carbohydrates and proteins compared to all investigated accessions and their control plants (Fig. 4c, d).

### Effect of salinity stress on the expression of glycine betaine (*CMO* and *BADH*) genes in *B. vulgaris* and *B. maritima*

**Fig. 5:**
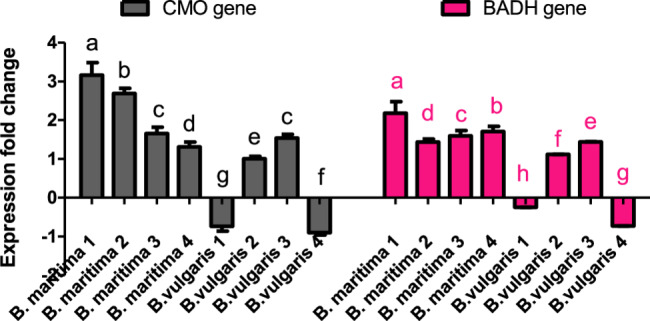
Effect of salinity stress (250 mM NaCl or 25 dS/m) on the gene expression of *CMO* and *BADH* genes of eight different of 70-day-old *B. maritima* (L.) and *B. vulgaris* (L.). Values represent means of 6 replicates ± standard error. Small identical letters on different columns indicate significant differences between treatments according to Duncan’s multiple-range test (*P* ≤ 0.05)

GB (*CMO* and *BADH*) gene transcripts in the leaves of *B. vulgaris* and *B. maritima* accessions were investigated in 10-week-old control and salt-stressed (250 mM NaCl) plants using primers specific to these two genes. The first gene encodes choline monooxygenase (*CMO*), while the second encodes betaine aldehyde dehydrogenase (*BADH*). The qRT-PCR results shown in Fig. 5 indicated that the expression of the *CMO* gene was significantly elevated in all *B. maritima* accessions 1, 2, 3, and 4 compared to the expression of the *GAPDH* gene by 3.17, 2.69, 1.65, and 1.31-fold, respectively. Additionally, *B. vulgaris* accessions 2 and 3 showed increases of 1.01- and 1.54-fold in *CMO* gene expression, respectively. In contrast, *B. vulgaris accessions* 1 and 4 exhibited slight downregulation of *CMO* gene expression by 0.75- and 0.9- fold, respectively (Fig. 5). The data illustrated in Fig. 5 show the pattern of *BADH* gene expression, which indicated a smaller increase in some accessions while demonstrating fairly similar fold changes in others when compared to *CMO* gene expression. According to our qRT-PCR data, *BADH* gene expression was significantly higher in all *B. maritima* accessions compared to their control plants, with increases of 2.19, 1.44, 1.59, and 1.71-fold in *B. maritima* accessions 1, 2, 3, and 4, respectively. Furthermore, *B. vulgaris* accessions 2 and 3 displayed increases of approximately 1.12- and 1.44-fold increases in *BADH* gene expression, respectively. However, in *B. vulgaris* accessions 1 and 4, *BADH* gene expression showed slight downregulation by 0.25- and 0.73-fold, respectively (Fig. 5).

### Effect of salinity stress on the content of secondary metabolites of *B. vulgaris* and *B. maritima*

**Fig. 6:**
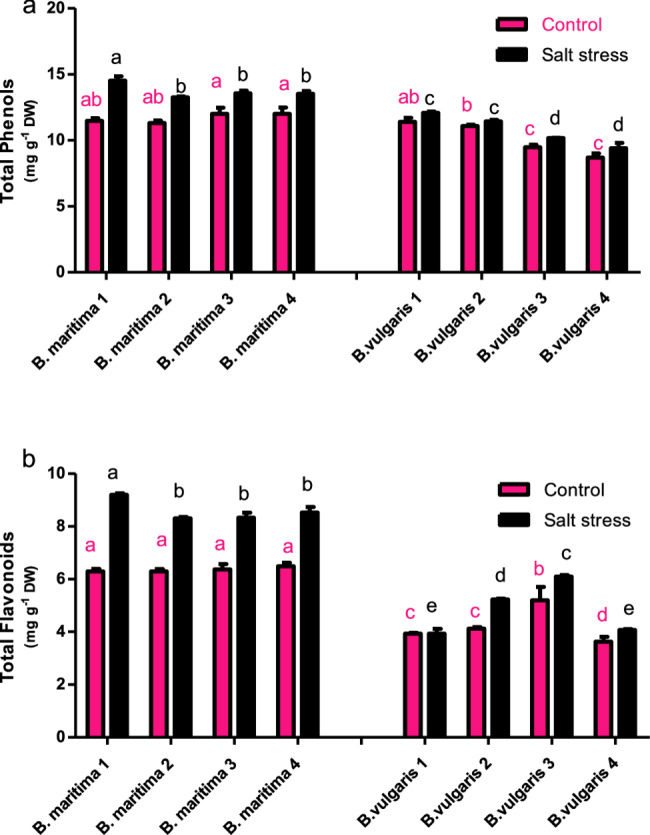
Effect of salinity stress (250 mM NaCl or 25 dS/m) on the content of total flavonoids (**a**) and total phenols (**b**) of eight different 70-day-old *B. maritima* (L.) and *B. vulgaris* (L.). Values represent means of 6 replicates ± standard error. Small identical letters on different columns indicate significant differences between treatments according to Duncan’s multiple-range test (*P* ≤ 0.05)

Data in Fig. 6 show the total phenol and flavonoid content in the shoots of the examined accessions in our study. The results indicated that all accessions of *B. vulgaris* and *B. maritima* salt-stressed plants had higher concentrations of flavonoid compounds after exposure to 250 mM NaCl compared to their control plants. Additionally, *B. maritima* accessions had a greater concentration of total phenols and total flavonoids than *B. vulgaris* accessions (Fig. 6). In contrast to their control plants, *B. vulgaris* accessions 1 and 4 exhibited lower percentages of flavonoids (0.08% and 11.9%), respectively, while *B. maritima* accessions 2 and 4 displayed higher flavonoid content (46.1% and 31.8%, respectively) than their control plants (Fig. 6a).

The results revealed that salinity stress had a non-significant effect on phenol content in all accessions investigated compared to control plants except for *B. maritima* 1 and *B. maritima* 2. The two accessions, *B. vulgaris* 1 and *B. vulgaris* 2, exhibited a lower percentage of phenol content due to salinity stress (6.14% and 3%, respectively), while *B. maritima* 1 and *B. maritima* 2 showed a higher percentage (26.7% and 17.4%, respectively) of phenol content (Fig. 6b).

### Effect of salinity stress on the content of minerals in *B. vulgaris* and *B. maritima*

**Fig. 7:**
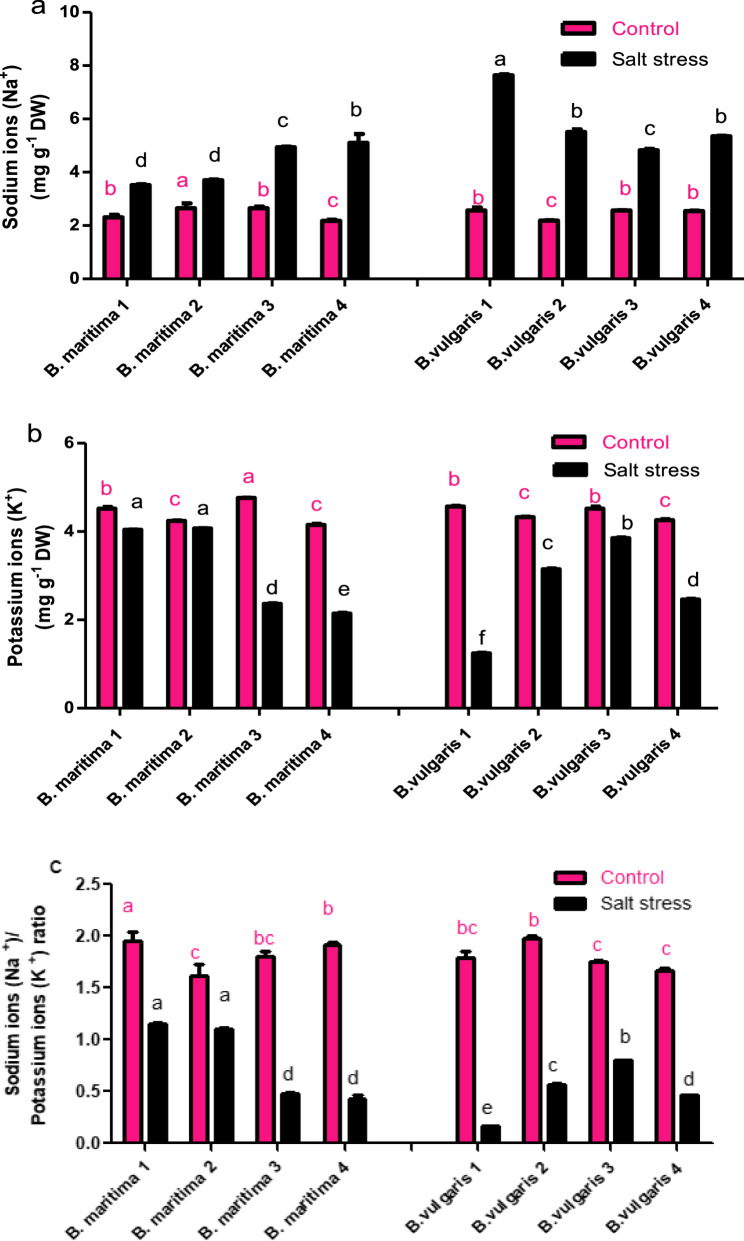
Effect of salinity stress (250 mM NaCl or 25 dS/m) on the content of sodium ions (Na^+^) (**a**), potassium ions (K^+^) (**b**), and ratio of potassium/sodium ions (K^+^/Na^+^) (**c**) of eight different of 70-day-old *B. maritima* (L.) and *B. vulgaris* (L.). Values represent means of 6 replicates ± standard error. Small identical letters on different columns indicate significant differences between treatments according to Duncan’s multiple-range test (*P* ≤ 0.05)

The findings presented in Fig. 7 demonstrate that all accessions of *B. vulgaris* and *B. maritima* exhibit highly significant changes in their Na^+^ and K^+^ concentrations in response to salinity stress. The concentration of Na^+^ in their leaves increases significantly in all tested accessions compared to their control plants. Additionally, the results indicated that the four *B. vulgaris* accessions under study had increases in Na^+^ ion concentrations of 197.14%, 151.98%, 87.93%, and 110.2% in *B. vulgaris* accessions 1, 2, 3, and 4, respectively. These percentages surpass those of the other four *B. maritima* accessions 1, 2, 3, and 4 by 51.87%, 39.74%, 86.16%, and 134.6%, respectively, indicating that *B. maritima* accessions are more salt-tolerant than the four *B. vulgaris* accessions. Furthermore, compared to their control plants, *B. vulgaris* 1 displays the highest increase, with a percentage of 197.14%. As shown in Fig. 7a, the *B. maritima* 1 and *B. maritima* 2 accessions have the lowest sodium concentrations, with percentages of 51.87% and 39.74%, respectively. In contrast, all accessions treated with salt experience a progressive drop in the concentration of K^+^ in their shoots compared to the control plants. It is evident that salt-stressed *B. maritima* 1 and *B. maritima* 2 exhibit higher concentrations of K^+^ than other stressed accessions, but their K^+^ percentages are 10.61% and 4% lower than those of respective control plants (Fig. 7b). Compared to control plants, the K^+^/Na^+^ ratio significantly decreases in all salt-stressed accessions due to changes in Na^+^ and K^+^ contents. *B. vulgaris* 1 has the lowest ratio, measuring at 0.16, while *B. maritima* 1 and *B. maritima* 2 have the highest ratios, measuring around 1.15 and 1.10, respectively (Fig. 7c).

### Effect of salinity stress on the tolerance index of *B. vulgaris* and *B. maritima*

The results shown in Table 2 indicate that the tolerance index of *B. vulgaris* and *B. maritima* exhibits varying responses to high salt stress, as assessed through morphological and biochemical parameters. The cultivated *B. vulgaris* accessions were found to have a lower tolerance to salt stress compared to the wild-type *B. maritima* accessions across all evaluated parameters. The tolerance was ranked from highest to lowest as follows: *B. maritima* 1 > *B. maritima* 2 > *B. maritima* 3 > *B. maritima* 4 > *B. vulgaris* 3 > *B. vulgaris* 2 > *B. vulgaris* 4 > *B. vulgaris* 1.

**Table 2:** Effect of salinity stress (250 mM NaCl or 25 dS/m) on the tolerance index (TI) of various parameters (SPAD values, fresh weight, dry weight, relative water content, proline, glycine betaine, sodium ions, potassium ions, flavonoid compounds, phenolic compounds, total soluble proteins, and total soluble carbohydrates) for eight different accessions of 70-day-old *B. maritima* (L.) and *B. vulgaris* (L.)

Tolerance Index (TI) (%)													
Accession	SPAD value (9-weeks)	SPAD value (10-weeks)	Fresh weight	Dry weight	Relative water content	Proline	Glycine betaine	Sodium ions	Potassium ions	Flavonoid compounds	Phenolic compounds	Total soluble proteins	Total soluble carbohydrates
*B. maritima* 1	−06.27	−25.85	−07.67	−20.67	−12.50	+ 208.50	+ 6.44	+ 51.87	−10.61	+ 46.10	+ 26.74	+ 42.85	+ 28.89
*B. maritima* 2	−03.06	−14.51	−18.68	−28.49	−29.64	+ 114.34	+ 9.29	+ 39.74	−04.00	+ 31.81	+ 17.40	+ 30.16	+ 25.90
*B. maritima* 3	−07.86	−19.91	−19.15	−32.12	−47.06	+ 128.03	+ 37.57	+ 86.16	−50.38	+ 30.82	+ 13.05	+ 27.93	+ 18.04
*B. maritima* 4	−23.77	−23.27	−26.46	−35.75	−57.99	+ 204.66	+ 34.94	+ 134.60	−48.39	+ 31.55	+ 12.77	+ 25.05	+ 17.79
*B. vulgaris* 1	−10.66	−41.91	−41.63	−71.24	−78.18	+ 41.47	−79.18	+ 197.14	−72.77	+ 0.084	+ 6.14	+ 3.34	+ 8.24
*B. vulgaris* 2	−10.47	−30.56	−19.18	−50.00	−66.67	+ 42.07	+ 31.30	+ 151.98	−27.25	+ 27.12	+ 3.00	+ 9.69	+ 13.91
*B. vulgaris* 3	−14.86	−40.68	−22.79	−30.42	−64.05	+ 114.27	+ 27.68	+ 87.93	−14.76	+ 17.30	+ 7.39	+ 19.17	+ 16.19
*B. vulgaris* 4	−07.28	−39.47	−13.17	−53.03	−72.16	+ 153.48	−47.36	+ 110.20	−42.17	+ 11.92	+ 8.04	+ 4.58	+ 6.94

Different letters indicate significant differences between treatments according to Duncan’s multiple-range test (*P* ≤ 0.05)

+TI means the percentage of increase in each parameter in response to different treatments in response to different treatments compared with control while

-TI means the percentage of decrease in each parameter in response to different treatments compared with control values. Values are means of 6 replicates ± SD

### Correlation analysis between morphological and biochemical characteristics and glycine betaine genes

**Fig. 8:**
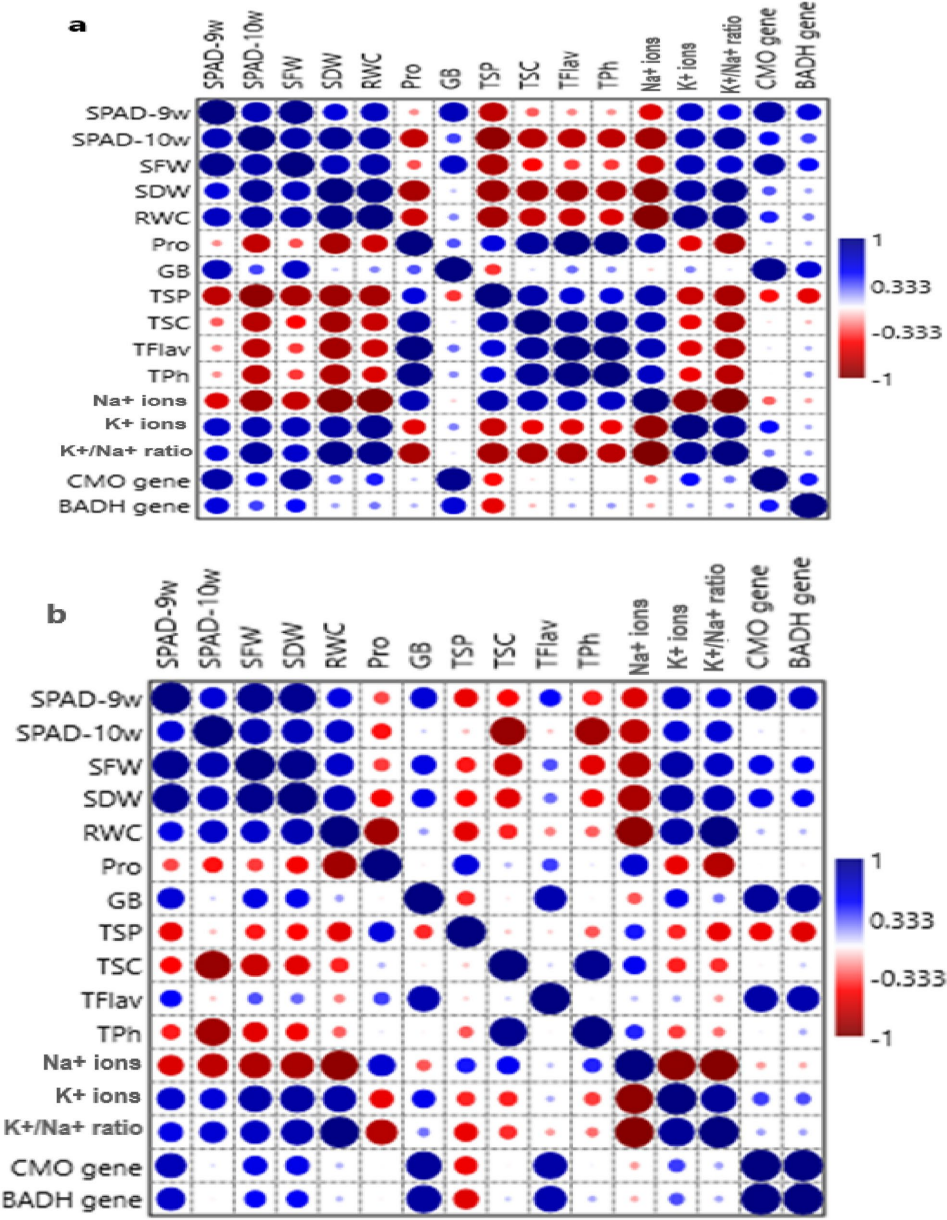
Heatmap of Pearson’s correlation analysis among all measured morphological and physiological parameters (SPAD-9w: SPAD value at 9 weeks old, SPAD-10w: SPAD value at 10 weeks old, SFW: shoot fresh weight, SDW: shoot dry weight, RWC: relative water content, Pro: proline, GB: glycine betaine, TSP: total soluble protein, TSC: total soluble carbohydrates, TFlav: total flavonoid, TPh: total phenols, Na^+^ ions: sodium ions, K^+^ions: potassium ions, K^+^/Na^+^ratio: ratio of potassium ions/sodium ions, *CMO* gene: choline monooxygenase gene and *BADH* gene: betaine aldehyde dehydrogenase gene for eight different accessions of 70-day-old salt-stressed *B. maritima* (L.) (**a**) and *B. vulgaris* (L.) (**b**)

Pearson’s correlation analysis (correlation coefficient; *r* = −1 to 1) was performed to determine the relationship among all measured morphological and physiological parameters and to evaluate the correlation between biosynthesized GB genes (*CMO* and *BADH*) and salinity stress in eight different accessions of 75-day-old salt-stressed *B. maritima* and salt-stressed *B. vulgaris* at *p* **≤** 0.05 (Table 3; Fig. 8). The data presented in Table 3; Fig. 8 reveal significant negative correlations between sodium ions (Na^+^) and chlorophyll content (SPAD value), shoot fresh weight (SFW), shoot dry weight (SDW), and relative water content (RWC). Furthermore, the results suggest that reducing salinity stress (Na^+^) significantly increased the content of GB and CMO and BADH as well as potassium ions (K^+^) (-ve correlation). In contrast, the levels of proline (Pro), total soluble proteins (TSP), total soluble carbohydrates (TSC), total phenols (TPh), and total flavonoids (TFlav) significantly increased in response to salinity stress (+ ve correlation) (Table 3; Fig. 8). Additionally, the data indicate a significant positive correlation between chlorophyll content and SFW, SDW, RWC, GB, and K^+^ ions across the eight different accessions of 75-day-old salt-stressed *B. maritima* and salt-stressed *B. vulgaris* at *p* **≤** 0.05 (Table 3; Fig. 8).

**Table 3:** Pearson’s correlation analysis between all measured morphological and physiological parameters (SPAD-9w: SPAD value at 9 weeks old, SPAD-10w: SPAD value at 10 weeks old, SFW: shoot fresh weight, SDW: shoot dry weight, RWC: relative water content, pro: proline, GB: glycine betaine, TSP: total soluble protein, TSC: total soluble carbohydrates, tflav: total flavonoid, tph: total phenols, na+^+^ ions: sodium ions, K^+^ ions: potassium ions, K^+^/Na^+^: ratio of potassium ions/sodium ions, *CMO* gene: choline monooxygenase and *BADH* gene: betaine aldehyde dehydrogenase of eight different accessions of 70-day-old salt-stressed *B. maritima* (L.) and *B. vulgaris* (L.)

**75-day old salt-stressed *B. maritima* (L.)**																
Parameters	SPAD-9w	SPAD-10w	SFW	SDW	RWC	Pro	GB	TSP	TSC	TFlav	TPh	Na^+^ ions	K^+^ ions	K^+^/Na^+^	CMO gene	BADH gene
SPAD-9w		0.019	0.0005	0.085	0.034	0.598*	0.024	0.029	0.437	0.562*	0.602*	0.096	0.047	0.106	0.008	0.016
SPAD-10w	0.790**		0.008	0.007	0.005	0.035	0.366	0.009	0.021	0.021	0.031	0.006	0.017	0.004	0.172	0.314
SFW	0.940**	0.841**		0.025	0.009	0.407	0.043	0.014	0.202	0.347	0.331	0.042	0.023	0.054	0.007	0.163
SDW	0.641*	0.902**	0.765**		0.0001	0.008	0.779**	0.004	0.007	0.009	0.011	0.0002	0.005	0.0002	0.413	0.627*
RWC	0.743**	0.863**	0.844**	0.906**		0.056	0.566*	0.004	0.044	0.0576	0.083	0.0001	0.0006	0.0002	0.262	0.519*
Pro	−0.239	−0.734**	−0.344	−0.853**	−0.693*		0.499	0.089	0.004	1.2491E-0	0.0001	0.017	0.113	0.010	0.777**	07401
GB	0.770**	0.302	0.720**	0.184	0.241	0.399		0.319	0.856**	0.483	0.583*	0.824**	0.549*	0.881**	0.0005	0.074
TSP	−0.751**	−0.966**	−0.832**	−0.856**	−0.847**	0.631*	−0.432		0.016	0.073**	0.090	0.011	0.045	0.009	0.223	0.122
TSC	−0.313	−0.741**	−0.502*	−0.887**	−0.711**	0.899**	0.076	0.823**		0.006	0.002	0.013	0.142	0.011	0.862**	0.740**
TFlav	−0.222	−0.713**	−0.385	−0.849**	−0.709**	0.990**	0.217	0.662*	0.897**		2.073E-05	0.017	0.109	0.011	0.883**	0.722**
TPh	−0.287	−0.726**	−0.399	−0.880**	−0.670*	0.984**	0.230	0.639*	0.870**	0.979**		0.037	0.152	0.026	0.989**	0.633*
Na^+^	−0.659*	−0.854**	−0.723**	−0.921**	−0.951**	0.815**	−0.0947	0.822**	0.807**	0.793**	0.737**		0.003	7.4792E-07	0.452	0.672*
K^+^	0.717**	0.799**	0.775**	0.860**	0.931**	−0.625*	0.254	−0.709**	−0.568*	−0.611*	−0.556*	−0.916**		0.001	0.218	0.710**
K^+^/Na^+^	0.6124*	0.878**	0.691*	0.955**	0.952**	−0.832**	0.064	−0.837**	−0.822**	−0.826**	−0.766**	−0.993**	0.914**		0.509*	0.652*
CMO gene	0.807**	0.534*	0.849**	0.337	0.454	0.115	0.943**	−0.485	−0.035	0.062	−0.005	−0.311	0.493	0.274		0.244
BADH gene	0.636*	0.373	0.502*	0.185	0.269	0.151	0.613*	−0.591	−0.143	0.150	0.200	−0.177	0.167	0.189	0.465	
**75-day old salt-stressed *B. vulgaris* (L.)**																
Parameters	SPAD-9w	SPAD-10w	SFW	SDW	RWC	Pro	GB	TSP	TSC	TFlav	TPh	Na^+^	K^+^	K^+^/Na^+^	CMO gene	BADH gene
SPAD-9w		0.001	0.0007	0.898**	0.105	0.383	0.074	0.138	0.200	0.192	0.179	0.090	0.054	0.113	0.002	0.045
SPAD-10w	0.921**		0.003	0.529*	0.010	0.077	0.203	0.074	0.298	0.535**	0.028	0.020	0.016	0.016	0.865**	0.954**
SFW	0.931**	0.876**		0.965**	0.049	0.349	0.110	0.253	0.0533	0.406	0.072	0.018	0.010	0.041	0.119	0.185
SDW	0.050*	−0.230	−0.018		0.086	0.017	0.196	0.292	0.853**	0.014	0.067	0.292	0.671	0.160	0.126	0.179
RWC	0.613**	0.837**	0.707**	−0.648*		0.005	0.650*	0.115	0.274	0.552*	1.4342E-05	0.0005	0.009	1.8654E-05	0.727**	0.745**
Pro	−0.359	−0.653*	−0.388	0.793**	−0.867*		0.929**	0.097	0.730**	0.353	0.014	0.060	0.133	0.020	0.991**	0.953**
GB	0.665*	0.537*	0.606*	0.512*	0.191	−0.078		0.298	0.909**	0.013	0.865**	0.426	0.138	0.512*	0.002	0.004
TSP	−0.527*	−0.659*	−0.474	0.421	−0.601*	0.635	−0.426		0.798**	0.835**	0.212	0.259	0.286	0.135	0.150	0.104
TSC	−0.566*	−0.413	−0.697*	0.074	−0.432	0.141	−0.059	−0.112		0.831**	0.235	0.158	0.273	0.304	0.954**	0.805**
TFlav	−0.536*	−0.289	−0.345	−0.810**	−0.249	0.376	0.814**	−0.081	0.091		0.394	0.764**	0.707**	0.655	0.008	0.012
TPh	−0.571*	−0.717**	−0.66*	0.673*	−0.982**	0.816	−0.072	0.494	0.476	0.350		0.0003	0.012	4.3453E-05	0.948**	0.701**
Na^+^ ions	−0.630*	−0.747**	−0.805**	0.427	−0.937**	0.679**	−0.323	0.460	0.552*	0.126	0.945**		0.0003	3.9298E-05	0.647*	0.704**
K^+^ ions	0.6968*	0.8036**	0.8327**	−0.1790	0.837**	−0.577*	0.571*	−0.430	−0.449	0.158	−0.820**	−0.950**		0.001	0.347	0.388
K^+^/Na^+^ratio	0.6032*	0.8025**	0.7258**	−0.5468*	0.9803**	−0.7877**	0.2734	−0.5730*	−0.4150	−0.1909	−0.9739**	−0.9747	0.902**		0.662*	0.677*
CMO gene	0.771**	0.071**	0.595*	0.586*	0.147	0.004	0.892**	−0.557	−0.024	0.843**	0.027	−0.192	0.384	0.183		2.5831E-06
BADH gene	0.716**	−0.029	0.520*	0.527*	0.137	−0.024	0.877**	−0.615	0.104	0.820**	0.162	−0.160	0.354	0.175	0.989**	

*Significant correlation at *p* ≤ 0.05

**Significant correlation at *p* ≤ 0.005

The color intensities of the heatmap (Fig. 8) indicate strong positive (blue) or negative (red) correlations. Areas with high Na⁺ concentration correlate with low levels of chlorophyll, fresh and dry weight, and moisture, appearing in red, which signifies a negative correlation. In contrast, regions with high values of proline, GB, or K⁺, along with the K⁺/Na⁺ ratio, displayed in blue, highlighting their roles in stress tolerance.

## Discussion

In this work, the photosynthetic capability of various *B. maritima* and *B. vulgaris* accessions under salt stress was measured based on the leaf’s SPAD value. After nine weeks of cultivation and two doses of salt stress, the SPAD value of *B. vulgaris* leaves changed significantly, and *B. maritima* was identified as the most tolerant cultivar when compared to *B. vulgaris*. After ten weeks of cultivation, the relative concentration of *B. maritima* chlorophyll gradually decreased as salt stress intensified. However, as the duration of NaCl salt stress increased, the difference in response between *B. vulgaris* accessions and *B. maritima* diminished over time. This indicates that *B. maritima* uses chlorophyll content regulation as a defense mechanism against NaCl stress and to maintain leaf function. Possible explanations include the following: Sodium stimulates the uptake of nitrate in *B. maritima* [30], total nitrogen concentration in plants increases with NaCl concentration [31], and there is a strong correlation between chlorophyll concentration and leaf N content because most leaf N is found in chlorophyll molecules [32].

One of the key physiological markers for assessing a plant’s ability to withstand stress is its chlorophyll concentration, which is directly correlated with the photosynthetic potential of crop leaves [33]. Consequently, the relative amount of chlorophyll was measured in this study using a SPAD chlorophyll meter. There are currently two prevailing schools of thought on the connection between salt stress and chlorophyll levels. The first asserts that low-sodium salt can increase the amount of chlorophyll in plants [34], while the second claims that salt stress elevates the activity of chlorophyll enzymes, exacerbates the breakdown of chloroplasts, and inhibits its production. According to Yin and Tian [35], the amount of chlorophyll and carotenoid pigments (photosynthetic pigments) rose in sugar beet leaves as salt stress increased.

In contrast to the control and other examined accessions (*B. maritima*), salt stress in this experiment resulted in a significant drop in the biomass of both fresh and dry shoots, as well as the relative water content (RWC) of *B. vulgaris* accessions. Reduced leaf growth and CO_2_ assimilation [36], altered RWC, and water potential in leaves [37], along with restricted leaf development, may contribute to beet growth deficits following salt stress. Although photosynthesis remains high levels even under elevated salinity, the observed growth reduction may be attributed to ion toxicity [38]. Salinity stress is one of the main abiotic factors limiting crop development and productivity. Globally, soil erosion, saline irrigation, climate change, and rising sea levels contribute to increased soil salinization, which has a detrimental effect on beet productivity [39, 40]. Even though beets are highly salt-tolerant plants, salinity stress factors lead to beet yield loss and stunt growth [41]. Therefore, it is imperative to fully understand plants’ physiological and genetic mechanisms for resisting abiotic stress [42]. Since leaves are organs involved in photosynthesis, their water status directly impacts photosynthesis, which in turn influences plant biomass and output. Plant leaves have evolved several adaptive modifications, such as reducing the number of new leaves produced and altering leaf shape, to decrease water loss [43].

According to our findings, *B. maritima* accessions exhibited a significantly higher proline content than those of the cultivated type (*B. vulgaris*) when exposed to salt stress. *B. maritima* (wild beet) is capable of accumulating proline in its tap roots when exposed to salt [15]. Salinity was consistently associated with an increase expression of the δ−1-pyrroline-5-carboxylate synthase (P5CS) gene in *B. maritima* which encodes the P5CS enzyme for proline synthesis [11]. In contrast, no significant increases in proline content were seen in *B. vulgaris* 1 or *B. vulgaris* 4. This implies that *B. vulgaris* accessions may no longer be able to sustain proline levels in high-salt environments. Small, non-toxic molecules known as compatible solutes or osmoprotectants help cells in withstanding stress by scavenging reactive oxygen species (ROS) and preserving the integrity of membrane and protein structures [44]. However, because Na^+^ plays a role in osmotic adjustment, low salt concentrations promote the development of beet plants [19].

It is well known that halophytic characteristics of beets are attributed to their capacity for osmotic adjustment, which is facilitated by accumulation of appropriate solutes in the cytoplasm and ions in the vacuoles of shoots in response to high osmotic pressure or salinity [45]. Proline is a crucial amino acid for plants as it helps regulate osmotic potential in response to a variety of environmental stressors, preserves membrane integrity, and stabilizes protein structures as a molecular chaperone [46]. Under salt stress, proline accumulation increased rapidly in several sugar, fodder, and red beet organs [11, 47, 48]. In addition, fodder beet revealed elevated proline levels in both taproots and leaves [11]. Furthermore, during salt stress, salt-tolerant beet accessions showed considerably higher proline levels than their sensitive counterparts [49, 50]. According to our findings, accessions *B. vulgaris* 1 and *B. vulgaris* 4 had less glycine betaine (GB) in their shoots under saline stress compared to other sensitive accessions, while the salt-tolerant *B. maritima* accessions had larger quantities of GB in their shoots. It was shown that GB, a nontoxic osmolyte and stabilizer of macromolecules, was considerably deposited in several organs of beet plants under salinity conditions [16, 51]. Increased GB accumulation has been observed in sugar beet roots [47] and leaves [51] under highly salinized circumstances. Notably, the osmotic adjustment under varying salt concentrations may be facilitated by sugar beet having greater GB levels compared to proline levels [52]. While GB inhibits the selective dissociation of extrinsic polypeptides from the PSII complex at high salt concentrations, salt stress disrupts the intermolecular interactions of protein subunits, specifically the D1 protein [53].

According to a related study conducted in *Synechococcus sp.*, GB attenuates the inhibitory effects of salt stress on D1 protein synthesis and degradation during photoinhibition [54]. Numerous investigations have revealed a substantial correlation between improved PSII photochemical performance and the improvement in photosynthesis that GB causes in salt-stressed plants [55–57]. In comparison to control and *B. vulgaris* accessions, our results demonstrated a highly significant increase in the levels of total soluble sugars and total soluble proteins in response to salt stress in *B. maritima* accessions. The production of antioxidant molecules and osmolytes may account for these outcomes in mitigating the detrimental effects of salinity stress. Under salt stress in beet genotypes, soluble sugars (sucrose, fructose, and glucose) may assist in maintaining cellular osmotic balance [49, 58]. Since sucrose was not reduced, it is essential for the stress response, osmotic adjustment, membrane and protein stability, and the prevention of protein denaturation [59]. As antioxidants and antiradicals, secondary metabolites play a crucial role in helping plants withstand oxidative stress [49]. Our findings showed that the expression of the *CMO* gene was considerably elevated in all *B. maritima* accessions compared to the expression of the *GAPDH* gene in the same accessions. According to reports by Surabhi and Rout [60], Omari Alzahrani [61], and Sugiharto et al. [62], the expression of the *COD* and *CDH* genes in a few additional plants exhibited similar protective effects on the photosynthetic apparatus under various abiotic stresses. In sugar beet genotypes with decreased salt tolerance, *BvCMO* and *BADH* gene expression levels were low [51]. Recent studies have shown that during salt stress, *BvCMO* gene expression was significantly upregulated in *B. maritima* guard cells and roots [63]. Tobacco plants that overexpressed the *B. maritima* *BvCMO* gene exhibited salt tolerance by increasing GB levels, as reported by Zhang et al. [64]. Additionally, *B. maritima* plants cultivated in high salinity demonstrated increased *BADH* gene expression, according to Skorupa et al. [15]. Nevertheless, increasing salinity in *B. vulgaris* did not result in enhanced transcription of this gene [6, 15].

The downregulation of *BADH* and *CMO* genes in accessions 1 and 4 indicates that each species has a unique response to salt stress. Consequently, the reduced activation of stress-responsive genes in certain *B. vulgaris* genotypes leads to lower glycine betaine accumulation. This results in slower growth and inadequate osmotic adjustment, negatively impacting tolerance and glycine betaine synthesis. Additionally, DNA methylation and histone modifications may act as epigenetic factors that restrict the response to salinity by inhibiting the expression of *BADH* and *CMO* genes. Moreover, the downregulation of these genes in *B. vulgaris* under salt stress affects the glycine-betaine pathway, suggesting diverse reactions to oxidative stress. Ultimately, the downregulation of *BADH* and *CMO* genes in accessions 1 and 4 points to a survival threshold under salt stress, implying that early adaptation is inadequate [51, 65].

These results suggest that wild beets outperform sugar beets in maintaining GB levels under salt stress. Conversely, the opposite effects were observed in sugar beets plants. For instance, Yolcu et al. [11] found that sugar beet roots and leaves expressed the *BADH* gene by 2 to 4-fold more than control plants at high doses of NaCl (greater than 200 mM), indicating the strong salt stress response of the *BvBADH* gene. This finding algins with our observations from qRT-PCR data, which showed that the expression of the *BADH* gene was significantly higher in all *B. maritima* accessions 1, 2, 3, and 4 compared to their control plants, by 2.19, 1.44, 1.59, and 1.71, respectively. In the genotype of salt-tolerant sugar beets, salt stress also elevated the expression of the *BvBADH7* gene [51]. Regardless of the source of osmotic stress, an increase in BADH transcripts was a common response, according to Xu et al. [66]. Another study by Moghaieb et al. [65] discovered that salt stress caused a rise in *BADH* mRNA levels at various salt concentrations (0, 85, 170, 340, and 510 mM), and that this expression was consistent with the betaine buildup observed. At the same NaCl levels, *S. europaea* plants actively accumulated more *BADH* mRNA than *S. maritima* plants, indicating that, similar to betaine accumulation in saline conditions, salt stress regulated BADH activity.

These outcomes are in line with research conducted on various other plants, including barley [67], sugar beet [68], and sorghum [69]. Choline availability is one of the primary factors that restrict GB accumulation in crop plants and accounts for some variation in GB production seen in the selected accessions of *B. vulgaris* and *B. maritima* [70]. Earlier research indicates that both endogenous and exogenous availability of choline can promote GB buildup [71]. The ability of various plant species to synthesize cytosolic choline and transfer it to the chloroplast for GB production is likely to vary. Similar to CMO-tobacco, COD-transgenic tomato plants accumulated more GB in the cytosol than in the chloroplast [72]. This suggests that the production of choline and/or its delivery to the chloroplast is less efficient in tobacco and tomato plants. Nevertheless, when exposed to salt stress, the amounts of GB accumulated in the cytosol and chloroplast of COD-transgenic rice and CDH-transgenic maize were equal. These findings indicate that the enhanced GB accumulation and ensuing stress tolerance in transgenic plants depend on both the species-specific availability of endogenous choline and its movement from the cytosol to the chloroplast [61]. This finding clarified how the *CMO* and *BADH* genes function in all *B. vulgaris* and *B. maritima* accessions under salt stress differed in terms of GB level.

Compared to control and *B. vulgaris* accessions, our results demonstrated a highly significant increase in the content of total phenols and flavonoids in response to salt stress in *B. maritima* accessions. The production of antioxidant molecules and osmolytes may be responsible for these outcomes, helping to mitigate the harmful effects of salinity stress. Phenolic compounds are produced in reaction to adverse environmental factors (such as light, cold, salt, etc.) and to protect wounded plants. They are essential for plant development and reproduction [11]. The study’s findings demonstrated that while vanillin and protocatechuic acid decreased under salty conditions, the concentration of total phenols and flavonoids rose in salt-tolerant accessions relative to the control under salt stress. Flavonoids with multiple hydroxyl groups are often more potent antioxidants against peroxyl radicals than phenolic acids and can also function as antioxidants against a range of readily oxidizable substances.

The results indicated that, compared to control and *B. maritima* accessions, salinity stress significantly increased the concentration of Na^+^ ions in the shoots of salt-sensitive *B. vulgaris* accessions. It also significantly lowered the K^+^ ion content and the K^+^/Na^+^ ratio in those same accessions. For halophytes to survive, plant cells must maintain low cytosolic sodium levels [45]. In response to salt stress, plant cells increase sodium levels, which is expelled from the plasma membrane and accumulates in the vacuole. Research has demonstrated that halophytes react to elevated NaCl salinity in diverse ways. Some plants that can tolerate salt exhibit a lower rate of Na^+^ and Cl^−^ -transport to their leaves compared to their salt-sensitive relatives [73]. To achieve low solute potentials and optimal growth, many halophytes even require high salt concentrations [18, 19]. According to Lv et al. [45], osmotic adjustment may be the most crucial aspect of beet salt tolerance. Additionally, numerous genes associated with the biosynthesis or transport of compatible solutes were identified in beets, and the impact of salt stress on their expression patterns was explored. Therefore, determinants of salt-tolerance can be considered the proteins and, eventually, the genes engaged in these processes. Intracellular sodium compartmentalization facilitates the plant salt tolerance [74], as evidenced by the cloning studies on Na^+^/H^+^ antiporters. Such compartmentalization of sodium and chloride can only be achieved through active transport into the leaf vacuole alongside limited tonoplast permeability to these ions.

## Materials and methods

### Plant material

The accession codes, geographic origins, and germination rates of eight accessions of both cultivated sugar beet *B. vulgaris* (L.) and wild beet *B. maritima* (L.) are presented in Table S1. In June 2022, seeds of all accessions were obtained from the Plant Breeding Institute at Christian-Albrechts-University (CAU-Kiel, Germany).

### Seeds germination and growth conditions

Seeds of *B. vulgaris* and *B. maritima* accessions were washed under tap water for three minutes, followed by cleaning with distilled water. The seeds were then surface sterilized for one minute using 70% ethanol, followed by twenty minutes in 2% sodium hypochlorite (NaOCl), and then rinsed with five times with sterilized deionized water. Sterilized seeds were planted in plastic pots (11 cm in height and 11 cm in diameter), each containing 1 kg of clay. Different concentrations of NaCl were prepared (0, 50, 100, 150, 200, 250, and 300 mM) to determine the lethal and sublethal concentrations of NaCl. The number of germinated seeds from various accessions of *B. vulgaris* and *B. maritima* was recorded, and the germination percentage was calculated after two weeks using the following equation:

$$\mathrm{Germination}\left(\%\right)=\frac{\mathrm{Number}\;\mathrm{of}\;\mathrm{germinated}\;\mathrm{seeds}}{\mathrm{Total}\;\mathrm{number}\;\mathrm{of}\;\mathrm{seeds}}\times100$$

Using six identical copies of each treatment, eight sets of pots were arranged under the same growth conditions in a randomized complete block design. The settings of the growth chamber were ideal 25°C/20°C (day/night), 65-75% relative humidity, and 8/16 hours of photoperiod (day/night). After a month of watering, the plants were trimmed so that there was only one plant in each container. The original electrical conductivity (EC) of the soil was determined (3.6 dS/m) using HI-8733 Reliable and Waterproof Multi-Range Conductivity Meters prior to salt treatment.

To determine EC, the nine-week-old plants were gradually watered with NaCl until the soil reached an initial EC of 25 dS/m was obtained. The plants were then watered again with NaCl for ten weeks, and shoot samples were collected in liquid nitrogen before being immediately frozen to isolate RNA. Leaf samples were selected from weeks 9 and 10 for analysis, corresponding to peak stress symptoms and late vegetative growth observed in preliminary trials.

### Growth parameters

The fresh shoot weights of *B. vulgaris* and *B. maritima* were measured in the growth chamber of the Plant Breeding Institute at CAU-Kiel, Germany. To get the turgid weights (TW), leaves were immersed in distilled water for eight hours following the measurement of the fresh weights (FW). To calculate the shoot dry weight (DW) (g), these tissues were dried in an oven at 70°C for a minimum of 72 h until they attained a consistent weight. Smart [75] provided the following formula to determine each plant’s relative water content:

$$\:RWC\:\left(\%\right)\:\:\frac{\left(FW\:-\:DW\right)}{\left(TW\:-\:DW\right)}\:\times100$$

The tolerance index (TI) for *B. maritima* and *B. vulgaris* was calculated using the formula by Turner and Marshall [76], which is as follows:

$$\mathrm{TI}=\frac{\mathrm{Mean}\;\mathrm{of}\;\mathrm{increase}\;\mathrm{or}\;\mathrm{decrease}\;\mathrm{of}\;\mathrm{the}\;\mathrm{measured}\;\mathrm{parameter}}{\mathrm{control}}$$

This index expresses all observable growth parameters (dry and fresh weights and relative water contents) as a comparative measure of tolerance to salt stress.

### Phytochemical analysis

The chlorophyll content of the plants was measured using an SPAD-502 chlorophyll meter (Minolta, Japan) after exposing the plants to two doses of NaCl (25 dS/m) for nine weeks and four doses of 250 mM NaCl for ten weeks. The SPAD value was used to indicate chlorophyll content. Glycine betaine (GB) content was determined using the periodide technique spectrophotometrically by reacting with iodine under acidic conditions and low temperatures to form the betaine–periodide complex, which appears as golden crystals and can be quantified by measuring optical density at 365 nm with GB as a standard curve [77, 78]. The concentrations employed for the calibration curve were (0, 150, 200, 250, and 300 µg ml^−1^) GB. Proline content was determined following the method described by Bates et al. [79], using a calibration curve of proline as the reference. The Bradford [80] method was used to determine the total soluble protein content using a calibration curve of bovine serum albumin as the reference, whereas Dubois et al. [81] method employed the phenol-sulfuric acid technique to estimate the overall amount of soluble carbohydrates. According to El-Shenody et al. [82], the concentration of total flavonoids and total phenols was determined. The contents of all osmolyte components were expressed in mg g^−1^ DW.

0.5 g of finely crushed dried leaves, 4 ml of nitric acid, and 2 ml of 30% perchloric acid were gradually heated until the mixture turned into a clear solution without burning, in accordance with the method described by Allen et al. [83]. Subsequently, the fluid was diluted to produce 50 milliliters. The concentrations of Na^+^ and K^+^ (mg g^−1^ DW) in the extract were measured using an Inductively Coupled Plasma Spectrometer (ICP/OES) (OPTIMA 8000, PerkinElmer, USA) at the Scientific Research Center and Measurements (SRCM) at Tanta University.

### Gene expression analysis of choline monooxygenase (*CMO*) and betaine aldehyde dehydrogenase (*BADH*) genes

There are two copies of the choline monooxygenase gene located on chromosomes 6 and 8. The gene on chromosome 6 is associated with the designed primer (Fig. S1). In contrast, the gene for betaine aldehyde dehydrogenase is found on chromosomes 1, 2, 5, and 7. The intended primer is linked to the gene on chromosome 5 gene (Fig. S1). Figure S1 was designed by the CLC Main Workbench 20.0.3 software tool, which is based on the alignment of genomic DNA sequence data from EL10.2 and the reference beet genome.

The CLC Main Workbench 20.0.3 software tool developed two primer combinations (one for each gene) of the GB group based on the alignment of genomic DNA sequence data from EL10.2 and the Reference Beet Genome. The German company EUROFINS MWG SYNTHESIS GMBH is where primer orders are placed. The primer sequences are shown in Table S2. Using the RNeasy Plant Mini Kit (Qiagen, Germany), RNA was extracted from eight accessions of *B. vulgaris* and *B. maritima* leaf samples for qRT-PCR analysis of CMO and BADH, collected on day 70 (week 10) after initiating salinity treatment. Next, the SensiFAST cDNA synthesis kit (Fisher Scientific, USA) was used to create cDNA. Primers BvCMO-F and BvCMO-R were employed for the *CMO* gene, while primers BvBADH-F and BvBADH-R were utilized for the *BADH* gene after routine PCR verification of the two proposed primer combinations with the generated cDNA. The reference gene *BvGAPDH* and SYBR green were used for qRT-PCR to determine the gene expression rate of the eight accessions. Using the ∆∆^Ct^ approach, the expression fold change of each gene correlated with the *BvGAPDH* expression rate under control and salinity conditions was calculated. The calculation and determination of the relative expression followed Livak and Schmittgen [84].

### Statistical analysis

All measurements were performed in six replicates, with data expressed as mean ± standard error (SE). A one-way analysis of variance (ANOVA) was conducted, followed by Duncan’s multiple range test for post-hoc comparisons among the treatment groups. Statistical differences were determined at a significance level of 0.05. Pearson’s correlation coefficients were calculated among variables using SPSS software (version 23) to interpret the relationships among treatments and measured parameters.

## Conclusions

This study demonstrates that salinity tolerance in *B. maritima* and *B. vulgaris* represents a complex, whole-plant response involving coordinated physiological, biochemical, and molecular mechanisms. Among the key determinants of salt tolerance are the abilities to maintain a favorable K⁺/Na⁺ ratio, effectively adjust osmotically through the accumulation of compatible solutes (proline, glycine betaine, soluble sugars, and proteins), and enhance the expression of genes related to glycine betaine biosynthesis (*BADH* and *CMO*). *B. maritima* accessions, particularly accessions 1 and 2, exhibited strong resilience under high salinity (250 mM NaCl), as evidenced by stable growth parameters, higher chlorophyll SPAD values, elevated levels of osmolytes and secondary metabolites, and upregulated expression of GB synthesis-associated genes (*CMO* and *BADH*). In contrast, *B. vulgaris* accession 1 was identified as the most salt-sensitive accession, showing downregulation of *BADH* and *CMO* genes, reduced osmolyte accumulation, and severe physiological impairment. These findings highlight the superior adaptive strategies of *B. maritima*, supporting its potential as a genetic resource for breeding salt-tolerant beet cultivars. Overall, salinity tolerance in these species is multifaceted, with the integration of ionic regulation, osmolyte synthesis, and stress-related gene expression emerging as the core of the tolerance mechanism.

## Supplementary Information

Supplementary Material 1.

## Data Availability

All data generated or analysed during this study are included in this published article and its supplementary information files.
