# Supplementary material for: Differential genetic and biochemical responses of Beta vulgaris and Beta maritima under salt stress
Source: BMC Plant Biol. 2025 Sep 2;25:1188. doi: 10.1186/s12870-025-07010-x (PMC12403458; doi:10.1186/s12870-025-07010-x)
Supplement: Supplementary file 1 — Supplementary Material 1. [file 12870_2025_7010_MOESM1_ESM.docx]

| 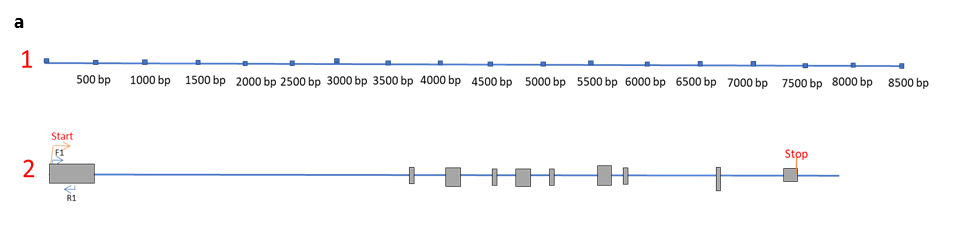  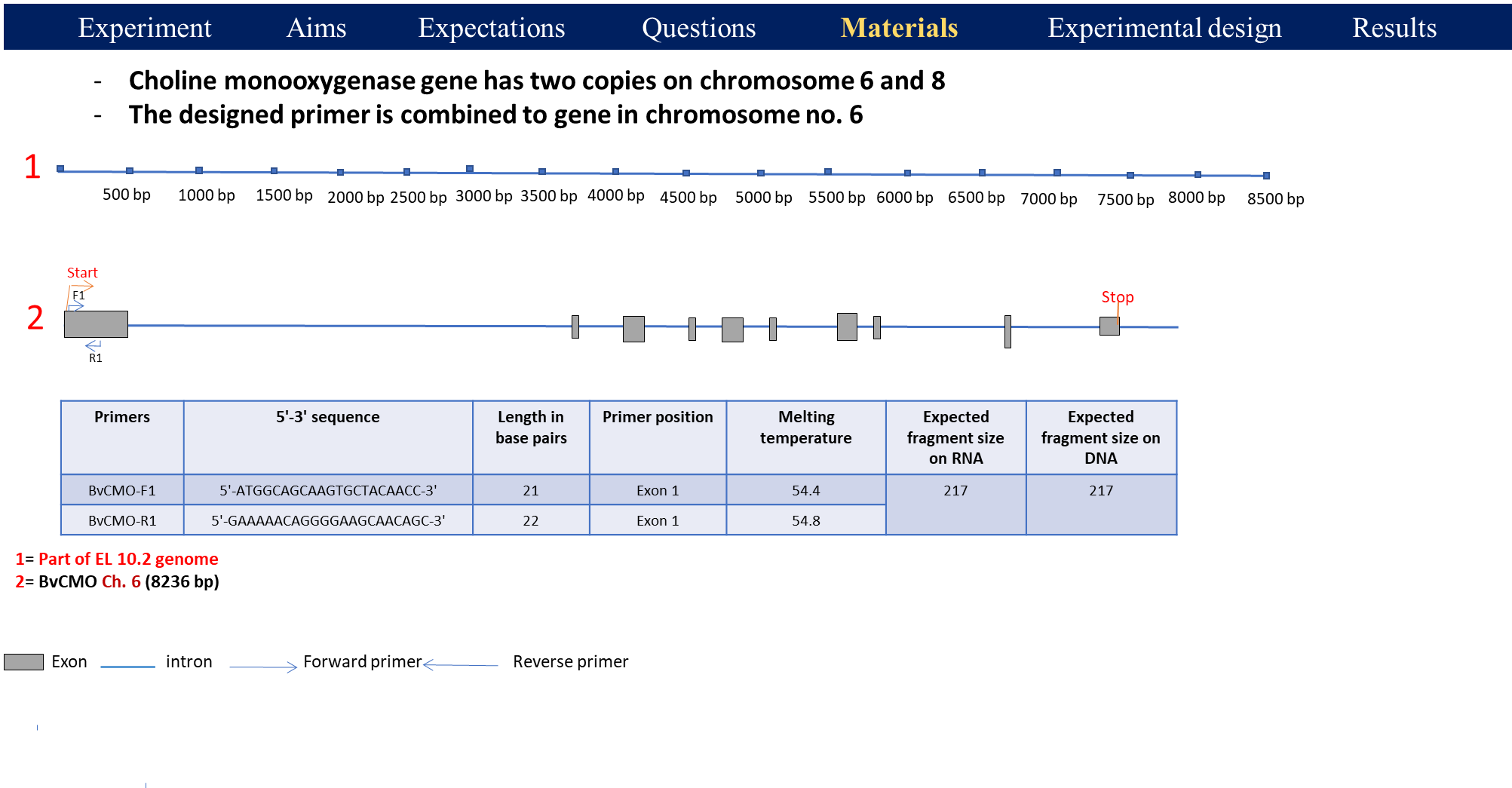 |
| --- |
| 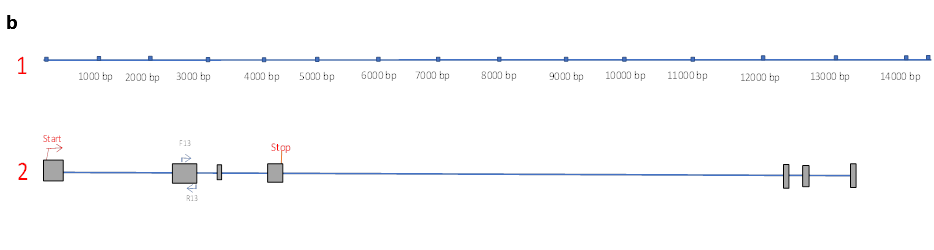   |

**Fig. S1:** Gene alignment of the *CMO* gene **(a)** and the *BADH* gene **(b)** based on genomic DNA sequence data designed by the CLC Main Workbench 20.0.3 software tool (QIAGEN).

**Table S1**: Seed accession codes, geographical distribution, and germination rates of *B. maritima* (L.) and *B. vulgaris* (L.).

| Accession code | Abbreviation | Crop type | Geographical origin | Germination rate | Number of seeds for 3 plants | Number of seeds for 6 plants |
| --- | --- | --- | --- | --- | --- | --- |
| 080279 | *B. maritima* 1 | Wild beet | Italy | 50 | 6 | 12 |
| 081683 | *B. maritima* 2 | Wild beet | North Wales | 10 | 30 | 60 |
| 205431 | *B. maritima* 3 | Wild beet | Greece | 30 | 10 | 20 |
| 960071 | *B. maritima* 4 | Wild beet | Italy | 140 | 3 | 6 |
| 093303 | *B. vulgaris* 1 | Sugar beet | USA | 30 | 10 | 20 |
| 080365 | *B. vulgaris* 2 | Sugar beet | Soviet Union | 80 | 4 | 8 |
| 080366 | *B. vulgaris* 3 | Sugar beet | Austria | 160 | 2 | 4 |
| 081898 | *B. vulgaris* 4 | Sugar beet | Turkey | 130 | 3 | 6 |

**Table S2:** Primer sequences, lengths, positions, melting temperatures, and fragment sizes of *CMO* and *BADH* genes based on genomic DNA sequence data from EL10.2 and the reference beet genome.

| Primers | Sequence (5`-3`) | Length (bp) | Primer position | Melting temperature | Expected fragment size on RNA | Expected fragment size on DNA |
| --- | --- | --- | --- | --- | --- | --- |
| BvCMO-F | 5`-ATGGCAGCAAGTGCTACAACC-3` | 21 | Exon 1 | 54.4 | 217 | 217 |
| BvCMO-R | 5`-GAAAAACAGGGGAAGCAACAGC-3` | 22 | Exon 1 | 54.8 |  |  |
| BvBADH-F | 5`-GCAACGTTCATGGATCATCTTA-3` | 22 | Exon 2 | 51.1 | 235 | 235 |
| BvBADH-R | 5`-AGCAATAAAGCCGATCCAAGC-3` | 21 | Exon 2 | 52.4 |  |  |
| BvGAPDH-F | 5`-CACCACCGATTACATGACATACA-3` | 23 | - | 61.5 | *Wetzel et al. (2021) | |
| BvGAPDH-R | 5`-GGATCTCCTCTGGGTTCCTG-3` | 20 | - | 62.0 |  |  |

* Wetzel V, Willems G, Darracq A, Galein Y, Liebe S, Varrelmann M. The *Beta vulgaris*-derived resistance gene Rz2 confers broad-spectrum resistance against soilborne sugar beet-infecting viruses from different families by recognizing triple gene block protein1. Mol Plant Pathol. 2021; 22(7):829-842. https://doi.org/10.1111/mpp.13066.
